# 

**Published:** 1969-01

**Authors:** 


### *Contents*

| | | Page |
|---|---|---|
| RICHARD SMITH JUNIOR AND HIS TIMES | *A. L. Eyre-Brook* | 1 |
| THE MANAGEMENT OF SPINA BIFIDA IN BRISTOL | *J. B. M. Roberts* | 12 |
| PAROXYSMAL TACHYCARDIA, EPILEPSY, FRAGILITAS OSSIUM AND MENTAL RETARDATION .......... | *J. Jancar* | 17 |
| THE SEVERAL AGES OF MEDICAL MAN | *Alan B. Raper* | 19 |
| NOTES AND NEWS .......................................................... | | 23 |
| BOOKS RECENTLY ADDED TO THE LIBRARY .............. | | 24 |

